# Vaginal Delivery vs. Cesarean Section: A Focused Ethnographic Study of Women’s Perceptions in The North of Iran

**Published:** 2015-01

**Authors:** Maryam Zakerihamidi, Robab Latifnejad Roudsari, Effat Merghati Khoei

**Affiliations:** 1 Department of Midwifery, School of Medicine, Tonekabon Branch, Islamic Azad University, Tonekabon, Iran;; 2 Evidence-Based Care Research Center, Department of Midwifery, School of Nursing and Midwifery, Mashhad University of Medical Sciences, Mashhad, Iran;; 3 Iranian National Center of Addiction Studies, Institution of Risk Behavior Reduction, Tehran University of Medical Sciences, Tehran, Iran

**Keywords:** Cesarean section, Ethnography, Natural childbirth, Pregnant women

## Abstract

**Background:** Cesarean section (C-section) in the North of Iran accounts for 70% of childbirths, which is higher than the national average of 55%. Understanding women’s perceptions towards modes of delivery in different cultures can pave the way for promoting programs and policies in support of vaginal delivery. We aimed to investigate women’s perceptions towards modes of delivery in the North of Iran.

**Methods:** Using a focused ethnographic approach and purposive sampling, 12 pregnant women, 10 women with childbirth experience, nine non-pregnant women, seven midwives, and seven gynecologists were selected from hospitals, healthcare centers, and clinics of Tonekabon and Chaloos, Mazandaran, Iran, during 2012-2014. Data were collected through in-depth interviews and participant observation. Data analysis was performed using thematic analysis using MAXqda software.

**Results:** Two major themes emerged from the data including: “vaginal delivery, a facilitator of women’s physical and mental health promotion”, and “C-section, a surgical intervention associated with decreased labor pain”. Six sub-themes subsumed within these major themes were: vaginal delivery as a safe mode of delivery, fullfilment of maternal instinct, a natural process with a pleasant ending, and C-section as a procedure associated with future complications, a surgical intervention and sometimes a life saving procedure, and a painless mode of delivery.

**Conclusion: **In the North of Iran, women’s justified cultural beliefs overshadow their micsconceptions, so it is hopped that through implementing appropriate training programs for raising awarness and correcting miscomceptions, vaginal delivery could be promoted even in regions with high rates of cesarean section.

## Introduction

Pregnancy is a physiological phenomenon, and its end is associated with pain, fear, anxiety, and even fear of death for mothers. Child delivery is a multi-dimensional process with physical, emotional, social, physiological, cultural, and psychological dimensions. Childbirth can be a critical and sometimes painful experience for women.1

Pain is one of the most common medical problems, which adversely affects an individual’s abilities and leads to fear and anxiety. Attitudes towards labor pain are associated with physical, psychological, environmental, and supporting factors, which greatly affect the decision about mode of delivery.2

Culture has a significant impact on people’s perceptions and attitudes towards labor pain, definition of labor pain, coping mechanisms against pain, and related behaviors. The attitude towards labor pain can be determinant of women’s decisions about mode of delivery.3

 One of the main goals of every medical team, dealing with childbirth, is performing a safe delivery. C-section was first introduced to reduce the risks for the mother and fetus. However, today, C-section is perceived as an escape from labor pain, and the false assumption that C-section is painless, safer, and healthier than vaginal delivery has become prevalent among women.3 In fact, more than half of the women voluntarily choose C-section as the preferred mode of delivery.4

During pregnancy, women should make a decision about the mode of delivery. Decision-making process is one of the most complex mechanisms of human thought, and is influenced by several factors. Decision-making is defined as “information analysis, making a decision, and implementing that decision”.^4^

The rate of C-section is one of the indices of health insurance. According to World Health Organization (WHO), C-section rate was reported as 15% in 1985. Based on the reports of WHO in 2009, this rate has significantly increased, worldwide.^5^ In the USA, C-section accounts for one in every 10 childbirths; in year 2002, C-section rate increased to 26.1% of all deliveries.^6^ Also, in European countries, this rate has been reported as 13-25%.^7^^,^^8^ In addition, in Latin American countries such as Brazil, rate of C-section is quite high (16.8 to 40%).^9^

In Iran, according to official statistics, prevalence of C-section is on average three times higher than the global rate; in fact, in years 2002 and 2003, it was estimated to be 36% and 33%, respectively.^10^

C-section is only recommended when the life of the mother or fetus is at risk. However, this method has currently become a way of escaping from labor pain. People have a common belief that cesarean delivery is less painful, safer, and healthier than vaginal delivery.^11^ In fact, more than half of women voluntarily undergo C-section.^12^ Individual’s views and attitudes signifcantly influence the choice of delivery. These views are based on different information sources, which are vary in terms of accuracy and reliability.^13^ Several studies have described a variety of factors for the selection of vaginal delivery. In the study of Black (2005) which was carried out in the UK, one of the most important determinants was the individual’s inclination towards vaginal delivery, which is influenced by several factors including interest in experiencing vaginal delivery, previous positive experiences, lack of anxiety about the safety of mother and baby, faster recovery after delivery, and fear of anesthesia.^14^

Also, in 2008, Kasai, in a qualitative study on women’s beliefs about mode of delivery in teaching hospitals of Brazil, showed that most women prioritized natural birth due to faster recovery after delivery. Also, the reason behind some women’s inclination towards cesarean section was lack of pain during labor and closing of the uterine tubes.^15^

Few qualitative studies have been carried out in Iran to investigate the conditions which influence women’s decision to choose either vaginal or cesarean delivery. In a focused ethnography conducted by Latifnejad Roudsari et al. (2014), fear of vaginal delivery, personal beliefs, cultural norms and values, and social network were reported as the factors affecting the choice of cesarean delivery.^16^

On the other hand, in a qualitative study by Zakerihamidi et al. (2014) it was reported that  economic issues, cultural beliefs and values, previous experiences of childbirth, significant others, and vaginal delivery facilitators were associated with the choice of vaginal delivery.^17^

It should be considered that sometimes mothers, who are not adequately informed about the mode of delivery, refuse to undergo C-section when this procedure is necessary for the prevention of maternal and fetal risks. As Aziken and colleagues (2007) reported that wrong cultural assumptions about delivery are the main reason for the mother’s refusal to undergo C-section, regardless of its necessity.^18^ So it seems that cultural norms and beliefs could affect an individual’s tendency towards a certain mode of delivery.

A review of the studies conducted in Iran indicates that except for a few qualitative studies, other articles had mostly adopted quantitative methods.  Also, the majority of studies were cross-sectional, evaluating different modes of delivery and the factors related to choosing a particular mode of childbirth. 

Therefore, there is insufficient knowledge about the perception and experiences of women on various modes of delivery. On the other hand, it is obvious that promotion of maternal health is not possible without a clear understanding of labor and women’s views on the related problems; therefore, effective interventions are required in accordance with the culture of a country or region. Given the high rate of C-section, the maternal and fetal risks associated with it, and lack of in-depth qualitative studies in Iran to describe the beliefs and perceptions of women about mode of delivery, it seems necessary to provide sufficient information about women’s attitudes towards vaginal delivery and cesarean section.

The main questions of this study were as follows:

1) What are the values, attitudes, beliefs, and perceptions of pregnant women, midwives, and gynecologists about different modes of delivery? 

2) What is the cultural meaning of childbirth from the perspective of mothers and health care providers? 

## Materials and Methods

Considering the subject of the present study, a focused ethnographic approach was applied. This method was selected since the researcher aimed to investigate the common behavioral patterns, attitudes, beliefs, and perceptions of participants about different modes of delivery. Therefore, it was necessary to evaluate the subjects’ interactions in the setting in order to identify the common patterns of behavior, attitudes, beliefs, and perceptions of the subjects. For this reason, focused ethnography was selected as the study method.

Focused ethnography evaluates a specific problem in a specific field with a small number of sample. The main features of this method are close observation of participants in the location, asking questions to gain an insight into current events, and using other available resources for a complete understanding of people, places, and events.^19^

Focused ethnography emphasizes on emic perspectives regarding specific activities and measures. In other words, in focused ethnography, it is not necessary to recognize the whole cultural background, but only certain elements of knowledge, related to the focus of the study, are targeted.^20^

Women’s beliefs and perceptions of vaginal delivery and C-section were thoroughly evaluated from 2012 to 2014 in order to facilitate a cultural understanding of delivery modes and finally, provide effective culture-based care strategies, based on their beliefs, perceptions, and views.

The study participants included 12 pregnant women in the third trimester of pregnancy, 10 women with childbirth experience, nine non-pregnant women, seven midwives, and seven gynecologists (a total of 45). The pregnant women were selected from those who were referring to healthcare centers or gynecology clinics of Tonekabon and Chaloos cities for prenatal care in the third trimester of pregnancy. Postpartum women were selected from those hospitalized in Tonekabon and Chaloos hospitals or those referring to healthcare centers or gynecology clinics to receive postpartum care (within six months after delivery). Non-pregnant women were chosen from native women, who were interested in participating in the study and had referred to healthcare centers or gynecology clinics of these two cities.

Exclusion criteria were as follows: 1) internal disorders and diseases; 2) obstetric complications, leading to emergency cesarean section; and 3) unwillingness to participate in the study.

The participants were selected using purposeful sampling and maximum variation strategy. The researcher selected the participants from different groups with different characteristics and points of view. The researcher introduced herself and explained the study objectvies to the participants. The subjects were ensured about the confidentiality of the data, and were able to withdraw from the study at any point.

By observing the ethical considerations, semi-structured interviews and observations were performed in a quiet and private environment by asking open-ended questions. 

The present study was approved by the ethics committee of Mashhad University of Medical Sciences, and written informed consents were obtained from all participants at the beginning of the study. The mean duration of interviews and observations was 1 hour and 1.5 hours, respectively for each participant. Observations were performed as “observer as participant” and were recorded as field notes in the study environment. 

In this study, during the observations, nine components of cultural context including environment of the study location, actors or participants in the settings, activities, objects, acts, events, time of activities, goals i.e. what people were aiming to do, and participants’ feelings were considered. 

In this study, the researcher fully immersed herself in the culture related to the selection of the mode of delivery in order to improve her interpretation and analysis of the topic under study. Therefore, she attended health care centers and gynecology clinics in Tonekabon and Chaloos for long periods and obsevered individuals working at health care centers or clinics, the services provided by them, the patients referring to these centers, and the interactions between pregnant women and midwives/gynecologists regarding the selection of the mode of delivery.

In addition to immersion in the data, the researcher recorded and reviewed her observations. The observations were recorded as field notes. The observation of participants not only led to an understading of the studied cultural field, but also helped the researcher to be a part of the culture, i.e., in addition to playing the role of an observer, the researcher was constantly presented in the setting as a participant. The combination of these two different roles (researcher as participant) contributed to the understanding of events and behaviors, related to the selection of the mode of delivery.^21^

To conduct semi-structured interviews, an interview guide was used. The questions were asked based on the individual’s answers and interview guide. In addition, during the rest of the interview, probing questions were used, if required.

In this study, after interviewing 45 participants, we reached data saturation. As the interviews and observations started, data analysis was simultaneously performed, using thematic analysis (Braun & Clarke, 2006), which included the following six steps: step 1, being familiarised with the data; step 2, forming initial codes; step 3, searching for themes; step 4, reviewing the themes; step 5, defining and naming the themes; and step 6, providing a report.^22^

At first, two primary interviews were conducted. The interview questions were concerned with the meaning and significance of vaginal delivery for the participant, the meaning and significance of C-section for the participant, the reasons for choosing vaginal delivery, and the reasons for choosing C-section. 

Some of the interview questions were related to the following issues: 

-How do you perceive vaginal delivery or cesarean section? 

-What are your beliefs about the mode of delivery? 

-What does vaginal delivery/cesarean section mean to you?

The most significant perceptions and views about vaginal delivery and C-section were categorized into 2 major themes and 6 sub-themes. 

In order to be ensured about the validity of the data, various steps were taken, as follows: long-term involvement with the participants during data collection; ongoing observations; triangulating different methods of data collection; providing in-depth descriptions; evaluation of the extracted codes and themes by experienced researchers (the second and third authors); and verification of the relevance of codes and themes by presenting the retrieved transcripts to the participants. 

## Results

The mean age of the participants was 25.19±4.68 years. Most of the subjects (80%) had high-school diplomas and the majority was housewives (70%); almost half of them (49%) were primiparous women. The most important points and views related to vaginal delivery and C-section were classified into two main themes: 1) vaginal delivery, a facilitator of women’s physical and mental health promotion, and 2) c–section, a surgical intervention associated with less labor pain. Also, six sub-themes including “vaginal delivery, a safe mode of delivery”, “vaginal delivery, elicitor of maternal feelings”, “vaginal delivery, a natural process with a pleasant ending”, “C-section, a procedure associated with future complications”, “C-section, a surgical intervention and sometimes a lifesaving procedure”, and “C-section, a painless mode of delivery” were derived from the data (Table 1).

**Table 1:** Themes and sub-themes emerged from the data

**Sub-themes**	**Themes **
Vaginal delivery, a safe mode of delivery	Vaginal delivery, a facilitator of women’s physical and mental health promotion
Vaginal delivery, fullfilment of maternal instinct	
Vaginal delivery, a natural process	
C-section, a procedure associated with future complications	C-section, a surgical intervention associated with decreased labor pain
C-section, a surgical intervention and sometimes a lifesaving procedure	
C-section, a painless mode of delivery	

*1. Vaginal Delivery, a Facilitator of Women’s Physical and Mental Health Promotion*

*-Vaginal Delivery, a Safe Mode of Delivery*

The participants’ understanding of the advantages of vaginal delivery and the few associated complications was among the most important positive perceptions about vaginal delivery. The participants believed that vaginal delivery led to minor complications and is not associated with problems of C-section such as back pain, pain/infection/irritation/or itching at the incision site, forgetfulness, death, or anesthesia-related complications. As one participant pointed out:

"*C-section may cause foot pain, back pain, digestive problems, asthma, abdominal pain, or back pain; but these complications do not happen in vaginal delivery.”* (Pregnant woman, 32 years old, high-school diploma, second pregnancy, experience of one C-section)

As some of the participants acknowledged, vaginal delivery was accompanied by fast recovery. As an interviewee said:

*“After natural delivery, I was able to do my daily tasks. I recovered pretty fast and helped others.”* (Potpartum woman, 25 years old, diploma, first pregnancy, experience of vaginal delivery)

"*In C-section, the baby is delivered while I’m under anesthesia. What’s the point? The pain starts after childbirth. But in vaginal delivery, pain is only before and during the delivery.”* (Pregnant woman, 30 years old, undergraduate student, first pregnancy, in favor of vaginal delivery)

As participants pointed out, vaginal delivery is a safe mode of delivery since it ensures the health of both mother and fetus and helps improve the health of family and community. One of the pregnant women stated that vaginal delivery detoxifies the body, and the body can regain its health:

"*Vaginal delivery is difficult, but all the toxins get out of your body, which is a good thing.”* (Pregnant woman, 28 years old, high-school diploma, first pregnancy, in favor of vaginal delivery)

A small number of interviewees believed that vaginal delivery leads to pelvic floor dysfunction, perineal relaxation, and orgasmic disorders; such information was provided by their friends and relatives. However, these participants still considered vaginal delivery as an acceptable mode of delivery with very few complications. As one participant mentioned:

A gynecologist, who avoided C-section due to non-medical reasons, in favor of vaginal delivery, noted the negative effects of pregnancy on pelvic floor muscles and mentioned the impact of pregnancy and hormonal changes on the loosening of pelvic floor muscles:

"*People think that vaginal delivery causes pelvic floor disorders, unlike cesarean section. I always tell mothers that pregnancy and its hormonal changes cause pelvic problems, regardless of the mode of delivery; this is why I prefer vaginal delivery.” *(A gynecologist, 52 years old, 15 years of work experience, experience of vaginal delivery)

*-Vaginal Delivery, Fullfilment of Maternal Instinct*

Considering the mother’s fast recovery and the few complications associated with vaginal delivery, the mother can regain her abilities to care for the child and play the maternal role. Therefore, she can establish an emotional relationship with her baby, and guarantee the infant’s mental health and even his/her social health in the future. The participants believed that only by performing vaginal delivery and enduring this difficult and exhausting experience, one can understand the mothers’ pain and great value. As one participant said:

"*The moment I gave birth to my baby, I appreciated my mother. In that very moment, I found out how great mothers are. This is why we can never repay what they have done for us.” *(A woman with previous experience of vaginal delivery, 26 years old, high-school diploma, first pregnancy)

Participants believed that during vaginal delivery, mothers actively participate in delivery and give birth after enduring excruciating pain. Therefore, maternal feelings are intensified by giving natural birth. As one participant pointed out:

"*When you hug your baby, it feels like God has given you an angel; a baby that you have given birth to.” *(Pregnant woman, 36 years old, MSc graduate, first pregnancy)

*-Vaginal Delivery, a Natural Process*

The results of this study showed that vaginal delivery is a symbol of joy and birth. What distinguishes labor pain from other types of pain is the pleasant ending, which makes vaginal delivery more acceptable. As an interviewee said:

"*Vaginal delivery is giving birth, is happiness and comfort. Although you feel so much pain, in the end, the pain ends in happiness. Vaginal delivery means birth; it is a good feeling.”* (Pregnant woman, 29 years old, bachelor’s degree, first pregnancy)

Most women believed that vaginal delivery is a natural, physiological phenomenon, during which no interventions are performed. They considered it a symbol of unification with nature and God’s creations; they believed that God helps them during labor. As one participant said:

"*Vaginal delivery is birth of a baby in the way that God intended to.”* (A participant with previous experience of vaginal delivery, 34 years old, MSc student, second pregnancy)

Some participants believed that vaginal delivery could help women attain a state of comfort. In fact, this feeling increases maternal satisfaction with vaginal delivery. This point can be summarized in an interviewee’s statement:

"*After the pain goes away, I feel happy for having passed this stage.” *(Postpartum mother, 31 years old, diploma, vaginal delivery)

*2. C-Section, a Surgical Intervention Associated with Decreased Labor Pain*

*-C-Section, a Procedure Associated with Future Complications*

Participants, in favor of vaginal delivery, believed that active maternal role during labor helps mothers form an enduring bond with their infants. They assumed that C-section deprives mothers of such feelings, since they do not experience the pain associated with vaginal delivery. As one participant remarked:

*"Those undergoing cesarean section can not take care of themselves or their babies. It is a bit difficult for them to regain their ability; they need more care.” *(Pospartum woman, 24 years old, diploma, first pregnancy, cesarean section)

Participants also mentioned some short- and long-term complications. The short-term complications include placental adhesion, inertia, hysterectomy, postoperative inability, leaving surgical instruments in the abdomen, anesthesia- and analgesia-related side effects, adverse gastrointestinal effects, several incisions on the body, postoperative pain, problems related to sutures, slow recovery, reduced lactation (resulting in formula use and colic).

Most interviewees with previous experience of C-section complained about the long-term complications including weight gain, uterine adhesions, uterine disorders, twinges at the incision site, pain at the incision site, swelling of the stomach, itching of the incision site, back pain, reduced mental security of the child, loss of concentration, and amnesia. In this regard, an interviewee said:        

"*I had amnesia after C-section. When I got home, it was like I had left the house for a long time. I didn’t remember where things were. I was very confused. Then, I found out it was a side-effect of general anesthesia.” *(Non-pregnant woman, 30 years old, bachelor’s degree, previous history of C-section)

Most participants believed that after one C-section, the next deliveries should be of the same nature. In other words, one C-section predicted the mode of next deliveries. As one participant said:

"*When you want to have another baby, you should have C-section again. When you have C-section for the first time, the next deliveries should be also the same.”* (Pregnant woman, 25 years old, high-school diploma, first pregnancy)

*-C-Section, a Surgical Intervention and Sometimes a Lifesaving Procedure*

From some midwives and physicians’ point of view, cesarean section was considered a type of intervention in the natural process. According to their belief, observing the process of natural childbirth and delivery of the fetus was a joyful experience, which helped physicians understand the power of God:

*“I do vaginal delivery, as well as cesarean section. Cesarean section does not give me the same feeling. I mean, the baby is delivered very quickly, but during natural delivery, the baby is born slowly, which is really enjoyable for me.”* (A gynecologist, 18 years of experience, in favor of vaginal delivery)

*-C-Section, a Painless Mode of Delivery*

Some pregnant women described C-section as”the last resort”. They beleived that in case of contraindications to vaginal delivery, performing C-section would be inevitable for the mother and fetus.**

*"In my opinion, C-section is the last resort. When there is absolutely nothing else that we can do and there is no other way to save the preganncy, we should choose C-section.”* (Pregnant woman, 26 years old, bachelor’s degree, first pregnancy, in favor of vaginal delivery)

According to some participants’ beliefs, C-section is less risky for the fetus, whereas vaginal delivery is only safe for the mother. They believed that C-section only poses risks for the mother. As one participant said: 

*“I just feel that C-section is less risky for my baby. Although it may cause some risks like infection for me, I am ok with it as long as my baby is safe.”* (Pregnant woman, 25 years old, high-school diploma, first pregnancy, in favor of C-section)

Pariticipants considered vaginal delivery as a painful experience, whereas C-section was assumed to be painless, due to receiving anesthesia. In many cases, mothers cannot tolerate the pain and they prefer C-section. In addition, fear and anxiety may lead to pain aggravation. As one participant remarked:

"*Because of the fear and pain of vaginal delivery, I want to have C-section. I think vaginal delivery is very difficult.”* (Pregnant woman, 24 years old, high-school diploma, first pregnancy, in favor of C-section)

One of the midwives, with a previous history of cesarean section, commented that pain after delivery in cases of cesarean section is mild and tolerable. Therefore, the mother is able to care for her baby and seems satisfied with cesarean section. She believed that pain after cesarean section is due to cutting multiple layers of the abdomen and uterus, which must be naturally greater than vaginal delivery, but the cause of decreased pain after cesarean section is due to routine use of diclofenac suppositories at the maternity unit of the hospitals: 

*“My pain reduced significantly. So I could take my baby to the hospital myself, when his skin had turned yellow; i could take care of my baby.” *(A midwife, 45 years old, bachelor’s degree, experience of cesarean section)

As some participants believed, the main advantages of C-section (compared to vaginal delivery) are preserving genital beauty, genital function, and sexual pleasure, and lack of cystocele, rectocele, and perineal relaxation. Physicians also had the same points of view. One interviewee said:

"*I knew that C-section has no pain and guarantees the health of my baby. It doesn’t lead to sexual dysfunctions either; so I wanted to have a C-section.”* (A participant with previous experience of C-section, 32 years old, bachelor’s  degree, in favor of C-section)

## Discussion

In the present study, the interviewees considered vaginal delivery as a natural phenomenon, since no interventions are required. In other studies, women described vaginal delivery as a natural process, and in case they were unable to undergo this procedure, they felt incompetent.^23^^-^^25^

Based on the findings of this study, vaginal delivery, given its particular nature and physical, psychological, and social advantages, is highly valued by most people. According to the interviewees’ statements, one of the reasons for mothers’ tendency towards vaginal delivery was their belief in the superiority of vaginal delivery, due to its positive outcomes for both mother and infant; the results were consistent with the findings of Fenwick et al.^24^ In some previous studies, participants believed that vaginal delivery was necessary for the baby’s lung development, improvement of mother-child emotional relationship, reduction of medication usage^24^^,^^25^ and other interventions such as epidurals and labor induction,^24^ and eliminating adverse labor-related outcomes; generally, they believed that vaginal delivery was less risky than c–section.^26^

In the present study, vaginal delivery was introduced as a manifestation of women’s power and ability to play the maternal role. The results of the current study considered vaginal delivery as an elicitor of maternal feelings. The findings were consistent with those of Fenwick and colleagues, who reported maternal/fetal health, mother-child communication, and transition to motherhood as the advantages of vaginal delivery.^24^

On the other hand, one of the problems, stated by women with cesarean experience and some pregnant women, was mothers’ inability to care for the child and fit the maternal role; the results were in accordance with those of Cranley and colleagues.^27^

The results of the present study demonstrated that vaginal delivery is a symbol of birth and happiness. The fact that childbirth pain is associated with positive outcomes distinguishes it from other sorts of pain. The study of Manthata also implicitly confirmed this finding as most subjects considered labor pain as a natural and necessary part of pregnancy.^28^

In the current research, participants considered vaginal delivery as a painful and fearful experience, while C-section was known as a pain-free, simple procedure, accompanied by anesthesia. Therefore, one of the main reasons for selecting C-section was fear of labor pain. The study of Poikkeus also showed that women’s preference for elective C-section is based on their unrealistic fear of pain during vaginal delivery and misconceptions about their inability to perform vaginal delivery.^29^

Despite the importance of these factors, limited studies have examined cultural beliefs about C-section,^30^^,^^31^ and very few of them have experimentally evaluated the impact of cultural beliefs on C-section. Therefore, further experimental studies are required to assess the direct relationship between cultural beliefs and tendency towards c–secttion (given the fear of labor pain).

Almost all pregnant women, who had a positive attitude towards C-section, attributed many advantages to this mode of delivery. The most important reason was lack of perineal relaxation, which had no negative impact on sexual activities. Gungor et al. (2008) in their study, also mentioned the same cultural belief. They found that sexual function and satisfaction are influenced by mental and emotional perceptions rather than physical factors; in fact, the frequency and mode of delivery are greatly influenced by cultural beliefs.^32^

Another important derived concept was “C-section as the last resort”. Petitti (1985) in his study believed that avoding C-section is associated with increased maternal/fetal morbidity and mortality, although some maternal deaths after C-section were directly related to surgical interventions; In fact, C-section can save the lives of mothers and babies in specific situations and emergencies.^33^

Participants in the present study mentioned the short-and long-term effects of C-section. In their opinion, one of the major complications of c–section is forgetfulness and memory loss, following the administration of anesthesia or analgesia; the obtained results were consistent with the results of Ryding et al. (2000).^34^

One of the strengths of this study was applying a qualitative approach for obtaining first-hand information about the assumptions and perceptions of women about vaginal delivery and C-section in Iran. In this regard, maximum variation in sampling was applied; i.e., different groups and places were selected for gathering the data.

In addition, lack of conducted research about women’s assumptions and views about vaginal delivery and C-section in Iran shows the importance of the current study. This study, by determining the viewpoints and beliefs of pregnant women about vaginal delivery and C-section, can greatly influence the cultural beliefs about C-section and reinforce positive attitudes towards vaginal delivery; therefore, individuals may be more inclined to choose vaginal delivery.

Lack of motivation in some participants for being interviewed or observed was one of the limitations of this study. In this study, perceptions of women about vaginal delivery and C-section were studied. However, further qualitative research is required to assess women’s decisions about the mode of delivery and describe their experiences of childbirth. 

## Conclusion

Pregnant women’s beliefs about vaginal delivery and C-section affect their decisions about the mode of delivery. The results showed that women in North of Iran believe that vaginal delivery, as a safe method, is not associated with complications occuring due to C-section. They also believe that natural birth has many benefits for the mother and baby.

The participants believed that labor pain enhances maternal feelings and strengthens mother-child relationship. In addition, vaginal delivery was considered a physiological process and a symbol of birth and happiness. 

On the other hand, most participants, who had positive perceptions about C-section and prioritized this mode of delivery, considered C-section as a painless and safe mode of delivery, which maintaine the beauty of reproductive organs. 

In order to develop a positive cultural and religious attitude towards vaginal delivery, awareness has to be raised through various ways, and the existing misconceptions should be corrected. Encouraging vaginal delivery among people as a mode of delivery which improves fetal/maternal health, raises women’s understanding of maternal identity, increases their comfort, and influences the decision concerning the mode of delivery via changing the existing beliefs and attitudes towards safe mode of delivery. 

## References

[1] JamshidiManesh M, Oskouie SF, Jouybary L, Sanagoo A (2009). The process of women’s decision making for selection of cesarean delivery. Iran Journal of Nursing.

[2] Farahani SM, Malekzadegan A, Mohammadi R, Hosseini F (2005). Effect of the one to one midwifery care during labor on modes of delivery. Iran Journal of Nursing.

[3] Lori JR (2009). Cultural Childbirth Practices, Beliefs and Traditions in Liberia.

[4] Adib Haj Bagheri M, Salsali M, Ahmadi F (2003). Clinical Decision-Making: a Way to Professional Empowerment in Nursing. Iranian Journal of Medical Education.

[5] Moore B (1985). Appropriate technology for birth. The Lancet.

[6] Finger C (2003). Caesarean section rates skyrocket in Brazil. The Lancet.

[7] McFarlin BL (2004). Elective cesarean birth: issues and ethics of an informed decision. Journal of Midwifery & Women’s Health.

[8] Saisto T, Halmesmäki E (2003). Fear of childbirth: a neglected dilemma. Acta Obstetricia et Gynecologica Scandinavica.

[9] Osis M, Padua K, Duarte G (2001). The opinion of Brazilian women regarding vaginal labor and cesarean section. International Journal of Gynecology & Obstetrics.

[10] Ahmad-Nia S, Delavar B, Eini-Zinab H (2009). Caesarean section in the Islamic Republic of Iran: prevalence and some sociodemographic correlates. Eastern Mediterranean Health Journal.

[11] Tatar M, Günalp S, Somunoğlu S, Demirol A, lu S, Demirol A (2000). Women’s perceptions of caesarean section: reflections from a Turkish teaching hospital. Social Science & Medicine.

[12] Gibbs RS (2008). Danforth’s obstetrics and gynecology.

[13] Faramarzi M, Pasha H, Bakhtiari A (2001). A survey on the knowledge and attitude of pregnant women to normal delivery in Babol, 1999. Journal of Babol University of Medical Sciences.

[14] Black C, Kaye JA, Jick H (2005). Cesarean delivery in the United Kingdom: time trends in the general practice research database. Obstetrics & Gynecology.

[15] Kasai KE, Nomura RM, Benute GR (2010). Women’s opinions about mode of birth in Brazil: a qualitative study in a public teaching hospital. Midwifery.

[16] Latifnejad Roudsari R, Zakerihamidi M, Merghati Khoei E (2015). Cultural perceptions and preferences of Iranian women regarding cesarean delivery. Iranian Journal of Nursing and Midwifery Research.

[17] Zakerihamidi M, Latifnejad Roudsari R, Merghati Khoei E (2015). Decision making for vaginal delivery in the North of Iran: a focused ethnography. Iranian Journal of Nursing and Midwifery Research.

[18] Aziken M, Omo-Aghoja L, Okonofua F (2007). Perceptions and attitudes of pregnant women towards caesarean section in urban Nigeria. Acta Obstetricia et Gynecologica Scandinavica.

[19] Speziale HS, Streubert HJ, Carpenter DR (2011). Qualitative research in nursing: Advancing the humanistic imperative.

[20] Knoblauch H (2005). Focused Ethnography. Forum: Qualitative Social Research.

[21] Roper JM, Shapira J (2000). Ethnography as method. In Methods in Nursing Research: Ethnography in nursing research.

[22] Braun V, Clarke V (2006). Using thematic analysis in psychology. Qualitative Research in Psychology.

[23] McGrath P, Phillips E, Vaughan G (2010). Vaginal birth after Caesarean risk decision-making: Australian findings on the mothers’ perspective. International Journal of Nursing Practice.

[24] Fenwick J, Gamble J, Hauck Y (2007). Believing in birth–choosing VBAC: the childbirth expectations of a self-selected cohort of Australian women. Journal of Clinical Nursing.

[25] Phillips E, McGrath P, Vaughan G (2009). ‘I wanted desperately to have a natural birth’: Mothers’ insights on Vaginal Birth After Caesarean (VBAC). Contemporary Nurse.

[26] Ridley RT, Davis PA, Bright JH, Sinclair D (2002). What influences a woman to choose vaginal birth after cesarean?. Journal of Obstetric, Gynecologic, & Neonatal Nursing.

[27] Cranley MS, Hedahl KJ, Pegg SH (1983). Women’s perceptions of vaginal and cesarean deliveries. Nursing Research.

[28] Manthata ALA, Hall DR, Steyn PS, Grove D (2006). The attitudes of two groups of South African women towards mode of delivery. International Journal of Gynecology & Obstetrics.

[29] Poikkeus P, Saisto T, Unkila-Kallio L (2006). Fear of childbirth and pregnancy-related anxiety in women conceiving with assisted reproduction. Obstetrics & Gynecology.

[30] Walker R, Turnbull D, Wilkinson C (2004). Increasing cesarean section rates: exploring the role of culture in an Australian community. Birth.

[31] Lo JC (2003). Patients’ attitudes vs. physicians’ determination: implications for cesarean sections. Social Science & Medicine.

[32] Gungor S, Baser I, Ceyhan T (2008). Original research–couples’sexual dysfunctions: does mode of delivery affect sexual functioning of the man partner?. The Journal of Sexual Medicine.

[33] Petitti DB (1985). Maternal mortality and morbidity in cesarean section. Clinical Obstetrics and Gynecology.

[34] Ryding E, Wijma K, Wijma B (2000). Emergency cesarean section: 25 women’s experiences. Journal of Reproductive and Infant Psychology.

